# Hyperuricemia and metabolic syndrome in type 2 diabetes mellitus patients at Hawassa university comprehensive specialized hospital, South West Ethiopia

**DOI:** 10.1186/s12902-017-0226-y

**Published:** 2017-12-12

**Authors:** Shiferaw Bekele Woyesa, Agete Tadewose Hirigo, Temesgen Bizuayehu Wube

**Affiliations:** 1 School of Medical Laboratory Sciences, Institute of Health Sciences, Jimma University, P.O. Box 378, Jimma, Ethiopia; 2 Hawasa University, Hawassa, Ethiopia

**Keywords:** Hyperuricemia, Metabolic syndrome, Hawassa, Ethiopia

## Abstract

**Background:**

Metabolic syndrome is a cluster of the most dangerous heart attack risk factors such as diabetes and prediabetes, abdominal obesity, high cholesterol and high blood pressure. Hyperuricemia is a condition in which the serum uric acid concentration is greater than 5.5 mg per deciliter for child and greater than 7.2 and 6.0 mg per deciliters for male and female adults respectively.

**Methods:**

A cross-sectional study was conducted to determine the magnitude of hyperuricemia and associated factors among type 2 diabetes mellitus patients at Hawassa Comprehensive Specialized Hospital (HCSH) from February 28 to May 30 /2017. A random sampling technique was used to include 319 study subjects and a signed consent had been provided by each study subject before running any data collection. An interviewer administered structured questionnaire was used to collect socio-demographic and some clinically useful data. In addition to this, we reviewed the records of the study subjects to obtain other useful clinical data. Five milliliter blood specimen was collected from each study subjects after overnight fasting. A25TM Bio-System Random Access chemistry analyzer was used for blood sample analysis. All data were checked visually, coded and entered into epi-data version 3.4 and statistical analysis was performed using SPSS version 20.0 software. Bi-variate and multivariate logistic regressions were used to determine the association between explanatory and the outcome variables.

**Results:**

The prevalence of hyperuricemia and metabolic syndrome among type 2 diabetic patients in the study area were 33.8%(*n* = 106) and 70.1% (*n* = 220) respectively. Having age greater or equal to 45 years (AOR: 1.9, CI: 1.-3.2, *P* value =0.015) and having metabolic syndrome (AOR: 2.6, CI: 1.5–4.7, *P* value = 0.001) were the determinant variables for hyperuricemia among type 2 diabetic patients.

**Conclusion:**

There was high prevalence of hyperuricemia among type 2 diabetic patients with high prevalence of metabolic syndrome. Therefore, regular health information about life style modification, early diagnosis and treatment for hyperuricemia and metabolic syndrome are essential to reduce hyperuricemia and metabolic syndrome in type 2 diabetic patients.

## Background

Diabetes is a chronic, progressive disease characterized by elevated levels of blood glucose. Type 2 diabetes mellitus (T2DM) results from the body’s ineffective use of insulin and it accounts for the vast majority of people with diabetes around the world. If diabetes is not well managed complications that may threaten and endanger life may develop [1–3]. Metabolic syndrome (MetS) consists of a constellation of metabolic abnormalities that confer increased risk of cardiovascular disease (CVD) and diabetes mellitus. The criteria for the metabolic syndrome have evolved since its original definition by the World Health Organization (WHO) in 1998. Central obesity, hypertriglyceridemia, low high-density lipoprotein cholesterol (HDL-C), hyperglycemia, and hypertension are the major risk factors for development of metabolic syndrome [4–7]. Insulin resistance is also correlated with risk factors of MetS such as dyslipidemia and hypertension. Moreover, insulin resistance has also a great impact on lipoprotein metabolism and it is associated with an increased level of triglycerides and reduced HDL-C in the blood. Some studies had revealed that there is a statistically significant association between hyperuricemia and insulin resistance was well demonstrated [8–12].

Hyperuricemia is a condition in which individuals have higher levels uric acid concentration in the serum or when serum levels of uric acid concentration is greater than the upper normal reference limits particularly greater than 5.5 mg per deciliter(mg/dl) for children and greater than 7.2 and 6.0 mg/dl respectively for both male and female adults. Uric acid is a final enzymatic product in the degradation of purine nucleotides and it has the ability to scavenge oxygen radicals and protect the erythrocyte membrane from lipid oxidation [13, 14]. It has been evidenced by certain studies that hyperuricemia is a risk factor for CVD in general population [15, 16] and studies conducted on laboratory animals also revealed that high concentration of uric acid played a causal role on MetS [17, 18]. Other Studies also indicated that an elevated level of uric acid predicts the development of diabetes, obesity, hypertension and the MetS [19]. A large epidemiological study done on adult men in Japan revealed that high uric acid serum concentration is a risk factor for T2DM. Even though studies reported hyperuricemia as a risk factor of T2DM, there are controversial ideas about the association between the two [20–23].

The main aim of the current study was to determine the prevalence of hyperuricemia and MetS in T2DM patients and associated risk factors. Furthermore, the study was also aimed to identify the correlation between biochemical measurements such as high density lipoprotein cholesterol, triglycerides, glucose and hyperuricemia.

## Methods

The aim of the current study was to determine the magnitude of hyperuricemia and metabolic syndrome and associated risk factors in T2DM patients. The Study was conducted at Hawasa University comprehensive specialized Hospital (HUCSH) from February 28 to May 30 /2017. HUCSH was established in November, 2006 and it is providing service for more than 15 million people of the Region. Currently, the hospital has over 400 beds and offers services at general and specialty level. There are 1323 registered T2DM patients at HUCSH, of which 1015 were T2DM and the remaining were type 1 diabetes mellitus. An institution based cross-sectional study design was implemented. All T2DM patients attending HUCSH diabetic clinic were source population. Those T2DM patients visiting HUCSH diabetic clinic during study period and selected based on inclusion criteria were considered as study subjects.

## Sample size determination and sampling techniques

A single population proportion formula was used to calculate the minimum sample size required and a random sampling technique was used to select 319 T2DM subjects by lottery method from 982 T2DM patients attending HUCSH. And those T2DM patients who had been taking blood lipid lowering therapy, pregnant, contraceptive users and history of hospitalization for thyroid disorders were excluded from the study.

## Data collection technique and instruments

### Socio-demographic, clinical and related data collection

Training was given for data collectors before any data collection. Interviewer administered structured questionnaire was developed for this study and used to collect all socio-demographic, and some clinical data. The medical record was reviewed to collect some secondary data. The data were collected by trained professional nurses working at diabetic clinic. Anthropometric data (weight and height) were collected according to WHO guideline manual [24]. Body mass index (BMI) was calculated weight divided by the square of height in kilogram per meters square formula (BMI = weight/height^2^ in kg/m^2^). Waist circumference (WC) was measured at the midpoint between the lower margin of the least palpable rib and the top of the iliac crest, using a stretch-resistant tape. WC was measured after instructing each study subject to stand with feet close together, arms at the side and body weight evenly distributed, wearing light clothes. The measurement was taken twice and the average was taken when the difference between the two measurements was within 1 cm and repeated measurements were taken when the difference between the two measurements was greater than 1 cm. Based on the WHO guideline manual of 2008 WC greater than 80 cm and 94 cm was considered abnormal for female and male respectively .

Systolic blood pressure (SBP) and diastolic blood pressure (DBP) was measured using mercury based sphygmomanometer after the subjects had rested for more than 10 min. For those study subjects with a SBP ≥ 140 mm of mercury (mmHg) and a DBP ≥ 90 mmHg, blood pressure was measured again and finally the average value was taken.

### Blood specimen collection and sample analysis

First five milliliter of venous blood was collected from each study subject after over night fasting. Secondly, the blood specimen was allowed to stay for 20–30 min for clot formation. Then, the specimen was centrifuged at 3000 revolution per minute (rpm) and the serum was separated from the whole blood. Finally, the serum was analyzed for HDL-C triglycerides (TGs), fasting blood sugar and uric acid by using A25™ Biosystem random access chemistry analyzer (linear chemicals, Montgat, Spain).

### Laboratory test method

Triglyceride (TG) was measured enzymatically in serum using a series of coupled reaction in which TG is hydrolyzed to produce glycerol. Glycerol is then oxidized to produce 4-(benzoquinone- monoimino)-phenazone that absorbs light and the amount of absorbed light that is directly proportional to the concentration of TG in the sample was measured spectrophotometrically at 500 nanometer (nm). HDL-C is measured directly in serum in which apo-B containing lipoproteins such as chylomicrons(CM), very low density lipoprotein(VLDL) and low density lipoprotein (LDL) in the specimen are reacted with a blocking reagent that makes them non-reactive with the enzymatic cholesterol reagent under conditions of the assay. As the result, the apo-B containing lipoproteins are effectively excluded from the assay and only HDL-C is detected under the assay condition.

Uric acid was also measured enzymatically in which uric acid is oxidized by uricase enzyme to produce allatoin and hydrogen peroxide. The hydrogen peroxide reacts with 4-aminoantipyrine and 3.5-dichloro-2-hydroxybenzene sulfonate in a reaction catalyzed by peroxidase enzyme to produce a colored product. The amount of light absorbed by the colored product which is directly proportional to the concentration of uric acid in the sample was measured spectrophotometrically at 520 nm.

Fasting blood sugar was measured enzymatically based on the glucose oxidase test principle in which glucose oxidase oxidizes beta D-glucose into D-gluconic acid and hydrogen peroxide. The hydrogen peroxide enters in to the second reaction involving p-hydroxybenzoic acid and 4-aminoantipyrine in the presence of peroxidase with the formation of a quinoneimine dye complex. This complex whose concentration is directly proportional to the concentration of glucose in the sample was measured spectrophotometrically at 510 nm.

### Data quality management

All collected data were checked for consistency and completeness daily by principal investigator. The pre-analytical, analytical and post analytical factors that can interfere to the results of biochemical measurements were controlled and maintained by medical laboratory technologist doing the specimen analysis. The proper functioning of instruments, laboratory reagents and technical performance were checked daily by using quality control samples before running patient samples and along with the patient samples and the run was repeated for the result fallen outside established values.

### Data processing and statistical analysis

All data were checked for consistency and completeness visually, and coded and entered into epidata version 3.4. SPSS version 20.0 software was used statistically for data analysis. Descriptive statistics like frequency and percentages were also used to describe data. Categorical variables were expressed as percentages. In addition, evaluation of differences in means of study groups was evaluated using student’s t-test and categorical variables was analyzed using chi-square tests and or fisher exact test. Pearson correlation coefficient was used to see the correlation between the dependent variable and MetS components. Bivariate and multivariate logistic regression models were used to assess statistically significant association between independent and dependent variables and those independent variables whose *P* value less than 0.25 in bivariate logistic regression model were moved to multivariate logistic regression to control possible confounder variables as well as to identify independent predictor variables that have statistically significant association with high concentration of uric acid (hyperuricemia). P value less than 0.05 in multivariate logistic regression was considered as statistically significant association.

### Limitation of the study

The study design was a cross-sectional which is referenced only about a single point in time. We did not also measure serum insulin concentration.

### Operational definitions

MetS: is defined according to National Cholesterol Education Program, Adult Treatment Panel (USNCEP-ATP) III guideline. Patients who have at least three of the following risk features to categorize in MetS: abdominal obesity (defined as WC > 102 cm in males and > 88 cm in females); elevated TGs (≥ 150 mg/dl); low HDL-C (< 40 mg/dl in males and < 50 mg/dl in females); elevated blood pressure and fasting blood sugar (FBS) > 110 mg/dl [25].

Raised Blood pressure or hypertensive: When systolic blood pressure is ≥ 130 or diastolic blood pressure is ≥ 85 mmHg according to national Cholesterol Education program (NCEP) definition criteria [25].

Hyperuricemia: Patients having serum uric acid levels more than 7.2 mg/dl in males and more than 6.0 mg/dl in females [26].

Regular physical activity: moderate physical activity such as walking, cycling or doing sport that has significant benefits for health [http://www.WHO.int/dietphysicalactivity/pa/en/.].

## Results

### Socio - demographic and clinical characteristics of the study subjects

A total of 319 study subjects were enrolled in this study and a 98.4% response rate was obtained. Majority, 67.0% (*n* = 211) of the study subjects were males and the remaining were females. The mean age of the study subjects was 49.8 ± 9.8 with the range of 30 to 80 years. Fifty one percent (*n* = 159), 88.5%(*n* = 278), 68.2%(*n* = 214) study subjects were urban dwellers, married, unemployed respectively. Majority, 82.5%(*n* = 259) study subjects travelled either by public transport or on foot. The educational status of about 40.4% (*n* = 127) study subjects was identified as greater or equal to secondary education. The prevalence of hyperuricemia and MetS were 33.8% (*n* = 106) and 70.1%(*n* = 220) respectively. The serum uric acid concentration was higher among male study subjects when compared to female (22.3% versus 11.5% respectively) and the prevalence was also higher among greater or equal to 45 years age group than age groups less than 45 years old (24.2% versus 9.6% respectively). The prevalence of hyperuricemia was also higher among study subjects with non-family history of diabetes as compared to study subjects with family history of diabetes (25.2% versus 8.6% respectively). Higher prevalence of serum uric acid concentration was determined among study subjects with less or equal to 10 year duration of diabetes as compared to the study subjects with greater duration of diabetes (26.4% versus 7.3% respectively). Moreover, higher serum uric acid concentration was determined among T2DM patients with MetS and among those with no history of regular exercise (27.7% and 24.8% respectively) as compared to those study subjects without MetS and with history of regular exercise (6.0% and 8.9% respectively).

The serum uric acid concentration had no significant difference among study subjects with body mass index (BMI) of 25–29.9 kg/m^2^ and greater or equal to 30 kg/m^2^ (6.4% versus 9.9% respectively). However, the highest prevalence of hyperuricemia, 16.6% (*n* = 52), was determined among overweight study subjects (BMI: 25–29.9 kg/m^2^) when compared to those study subjects with under (< 18.4 kg/m^2^) or normal (18.5–24.9 kg/m^2^) body weight and central obesity (≥ 30 kg/m^2^). There was no significant difference in serum uric acid concentration between non-hypertensive and hypertensive T2DM patients (19.7% versus 14.0% respectively) (Table 1).

**Table 1:** Socio-demographic and clinical characteristics of study subjects (*n* = 314)

Variables	Category		Uric acid level		Mean ± SD	*P*-value
		N (%)	Hyper uricemia(%)	Normo uricaemia(%)		
Sex	Male	211(67.2)	70(22.3)	141(44.9)		0.76
	Female	103(32.8)	36(11.5)	67(21.3)		
Age (in years)	< 45	120(38.2)	30(9.6)	90(28.6)	49.8 ± 9.8	0.01
	≥ 45	194(61.8)	76(24.2)	118(37.6)		
FHD	No	259(82.5)	79(25.2)	180(57.3)	36.1 ± 8.6	0.007
	Yes	55(17.5)	27(8.6)	28(8.9)		
DDM (years)	≤ 10	248(79.0)	83(26.4)	165(52.5)	7.21 ± 7.4	0.83
	> 10	66(21.0)	23(7.3)	43(13.7)		
Regular exercise	Yes	100(31.8)	28(8.9)	72(22.9)	1.5 ± 0.5	0.14
	No	214(68.2)	78(24.8)	136(43.3)		
MetS	Yes	220(70.1)	87(27.7)	133(42.4)	26.4 ± 10.1	0.001
	No	94(29.9)	19(6)	75(23.9)		
BMI (Kg/m^2^)	< 18.4	11(3.5)	3(2.8)	8(3.8)	25.6 ± 4.3	0.0001
	18.5–24.9	130(41.4)	20(6.4)	110(52.9)		
	25–29.9	114(36.3)	52(16.6)	62(29.8)		
	≥ 30	59(18.8)	31(9.9)	28(13.5)		
High blood pressure	Yes	80(25.5)	44(14.0)	36(11.5)	101.4 ± 11.8	< 0.0001
	No	234(74.5)	62(19.7)	172(54.8)		

FHD-family history of DM; BMI, body mass index; DDM, duration of diabetes mellitus

### Characteristics of MetS components

From 33.8% (*n* = 106) hyperuremic T2DM patients, about 27.7%(*n* = 87) of them had MetS. This indicates that the prevalence of MetS is significantly high among type 2 diabetic patients with abnormal serum uric acid concentration. There was positive correlation between high prevalence of MetS, 70.1%(*n* = 220) and abnormal serum uric acid concentration, 27.7%(n = 87) in T2DM patients (*P* value =0.001). Abnormal serum uric acid concentration was determined among 12.7%(*n* = 40) study subjects with both central obesity and higher diastolic blood pressure (*P* value = 0.0001 and 0.006 respectively) and among 15.6%(*n* = 49) study subjects with higher systolic blood pressure (P value = 0.0001). Hpertriglyceridemia, reduced HDL-C and hyperglycemia were determined among 70.4%(*n* = 221), 39.2%(*n* = 123) and 80.0% (*n* = 252) study subjects respectively. The abnormal serum uric acid concentration was determined among 31.2%(*n* = 98) of study subjects with hypertriglyceridemia (*P* value = 0.0001) and among 12.1%(*n* = 38) study subjects with reduced HDL-C9 *P* value = 0.4) and in 29.3%(*n* = 92) subjects with hyperglycemic (*P* value =0.04) (Table 2).

**Table 2:** Prevalence of MetS and its components among T2DM patients in relation to hyperuricemia (*n* = 314)

Variable	N (%)	Hyperuricemia N (%)	Normouricaemia N (%)	Mean ± SD	*P*-value
MetS	220(70.1)	87(27.7)	133(42.4)	26.4 ± 10.1	0.001
WC ≥ 102 for male, ≥ 88 cm for female	193(61.3)	40(12.7)	153(48.7)	93.6 ± 18.1	0.0001
TG ≥ 150 mg/dl	221(70.4)	98(31.2)	123(39.2)	202.5 ± 89.8	0.0001
Low HDL-C, (< 40 mg/dl for males, < 50 mg/dl for females)	123(39.2)	38(12.1)	85(27.1)	54.9 ± 59.2	0.4
FBS > 110 mg/dl	252(80.0)	92(29.3)	160(50.6)	200 ± 100	0.04
SBP > 130 mmHg	85(27.1)	49(15.6)	36(11.5)	123.6 ± 14.3	0.0001
DBP > 85 mmHg	88(28.0)	40(12.7)	48(15.3)	79.2 ± 9.2	0.006

BMI:body mass index; FBS:fasting blood sugar; HDL-C:high density lipoprotein cholesterol,; WC: waist circumference

The person correlation coefficient had indicated a positive correlation between abnormal serum uric acid concentration and all MetS components except HDL-C (Table 3).

**Table 3:** Pearson correlation coefficients of parameters of MetS with uric acid level (*n* = 314)

Parameter	Correlation coefficient	Mean ± SD	*P*-value
HDL-c	−0.05	54.9 ± 59.2	0.4
SBP	0.3	123.6 ± 14.3	< 0.0001
DBP	0.2	79.2 ± 9.2	0.006
WC	0.4	93.6 ± 18.1	< 0.0001
TG	0.4	202.5 ± 89.8	< 0.0001
FBS	0.1	200 ± 100	0.5

HDL-C:high density lipoprotein; TG: triglycerides; SBP-Systolic blood pressure; DBP:diastolic blood pressure; WC- waist circumference; FBS- fasting blood sugar

### Factors associated to hyperuricemia

Independent variables whose *P* value less than 0.25 in binary logistic regression model were shifted into multivariate analysis model to identify independent predictor variables for abnormal serum uric acid concentration or hyperuricemia. According to this statistical analysis, T2DM patients with age greater or equal to 45 years old were about 1.9 times (AOR = 1.9, C.*I* = 1.2–3.2, *P* value < 0.05) more likely to develop hyperuricemia when compared to those with less than 45 years old. T2DM patients with MetS were about 2.6 times (AOR = 2.6, C.*I* = 1.5–4.7, *P* value < 0.05) more likely to develop hyperuricemia when compared to those without MetS. Those study subjects with history of alcohol consumption during the study period were about 1.6 times (COR = 1.6, C.*I* = 0.8–3.4, *P* value = 0.26) more likely to develop hyperuricemia than those without history of alcohol consumption. Similarly study subjects with history of cigarette smoking were about 1.1 times (COR = 1.1, C.*I* = 0.5–2.4, P value > 0.05) more likely to develop hyperuricemia than those who did not smoke and also those who did not perform regular exercise were about 1.5 times (AOR = 1.5, C.*I* = 1.33–2.45, *P* value > 0.05) more chance to develop hyperuricemia than those who had been performing regular exercise (Table 4).

**Table 4:** Binary and multiple logistic regression in T2DM patients (*n* = 314)

Variables	Category	Uric acid level		COR (95%CI)	*P* value	AOR (95%CI)	*P*-value
		Hyper uricemia	Normouricaemia				
Sex	Female	36(11.5)	67(21.3)	1	0.8		
	Male	70(22.3)	141(44.9)	0.9(0.56–1.52)			
Age	< 45	30(9.6)	90(28.6)	1		1	0.015
	≥ 45	76(24.2)	118(37.6)	1.9(1.2–3.2)*	0.01	1.9(1.1–3.2)**	
Residence	Urban	59(18.8)	100(31.8)	1		1	0.5
	Rural	47(44.3)	108(51.9)	1.3(0.9–2.2)*	0.20	0.8(0.5–1.3)	
Occupation	Unemployed	71(22.6)	143(45.5)	1	0.8		
	Employed	35(11.1)	65(20.7)	1.1(0.7–1.8)			
Smoking	No	94(29.9)	187(59.6)	1	0.7		
	Yes	12(3.8)	21(6.7)	1.1(0.5–2.4)			
Alcohol consumption	No	92(29.3)	190(60.5)	1	0.26		
	Yes	14(4.5)	18(5.7)	1.60(0.8–3.4)			
Regular exercise	No	78(24.8)	136(43.3)	0.14(0.12–1.8)	0.22	1.5(1.33–2.45)	0.21
	Yes	28(8.9)	72(22.9)	1		1	
Duration of DM(years)	≤ 10 years	83(26.4)	165(52.5)	1	0.8		
	> 10 years	23(7.3)	43(13.7)	1.1(0.6–1.9)			
MetS	No	19(6)	75(23.9)	1	0.001	1	0.001
	Yes	87(27.7)	133(42.4)	2.6(1.5–4.7)*		2.6(1.5–4.6)**	

Note: *p < 0.25 for COR, **P < 0.05 for AOR; COR: Crude odds ratio; AOR: Adjusted odds ratio; MetS: metabolic syndrome

## Discussion

Although the prevalence of hyperuricema is high in T2DM patients, there is a controversial idea about the association between hyperuricemia and T2DM. We assessed the magnitude of hyperuricemia and MetS in T2DM patients and identified 33.8% of the study subjects were hyperuremic and the prevalence of MetS in study subjects was 70.1%. The prevalence of hyperuricemia reported from Indore [27] is comparable to the current finding of hyperuricemia. In contrast to the current finding, less prevalence of hyperuricemia was reported by Mundhe et al. from India [1] and much less prevalence was also reported from Nigeria [3].

Again the prevalence of hyperuricemia reported from China [28] is comparable to the current finding, however the prevalence of hyperuricemia was high among female study subjects in case of the finding reported from China in contrast to our finding in which male study subjects were more hyperuremic than female. Male study subjects were about two times more hyperuremic than females and study subjects whose age group greater or equal to 45 years were almost three times more hyperuremic than the others. Study subjects with family history of diabetes were less hyperuremic when compared to those with study subjects without family history of diabetes and study subjects with less or equal to 10 year duration of diabetes were three times more hyperuremic than other study subjects with longer duration of diabetes. Again the prevalence of hyperuricemia was high among study subjects with MetS. Similar findings were reported from Nigeria [29] and India [30].

National Cholesterol Education Program- Adult treatment panel III (NCEP-ATP III) was used to determine the prevalence of MetS in the current study. According to NCEP-ATPIII, the prevalence of MetS in current study was higher than the finding reported from Cameroon by similar criteria [31] and much higher than the finding reported from Ghana [32] by Mogre et al. and from Thailand [16] by Jaipakdee et al. Unlike the study reported from China [28], clinical parameters like high BMI, hypertension, WC and fasting blood sugar as well as biochemical parameters like TG and low HDL-C were not identified as risk factors for high prevalence hyperuricemia in current study. Duration and family history of diabetes had no statistically significant association with high prevalence of hyperuricemia in contrast to the finding reported from India [33].

Age greater or equal to 45 years and MetS were the only two parameters identified as independent predicator variables for high prevalence of hyperuricemia in T2DM patients. Clinical parameters like high BMI, hypertension, WC, fasting blood sugar and biochemical parameters like TG and low HDL-C were not identified as risk factors for hyperuricemia unlike the study reported from China [28].

## Conclusions

High prevalence of hyperuricemia and MetS was determined in T2DM patients. Both MetS and age greater or equal to 45 years were identified as independent predictor variables for the high prevalence of hyperuricemia in T2DM patients. Hypertriglyceridemia, hyperglycemia and low HDL-C had positive correlation with the high prevalence of hyperuricemia in T2DM patients.

## Abbreviations

ATP: Adult treatment Panel
BMI: Body Mass Index
CVD: Cardiovascular diseases
DBP: Diastolic blood pressure
FBS: Fasting blood Sugar
HDL-C: High density lipoprotein cholesterol
HUCSH: Hawassa University comprehensive specialized Hospital
IDF: International Diabetic Federation
LDL-C: Low density lipoprotein cholesterol
MetS: Metabolic syndrome
NCEP: National cholesterol Education program Adult treatment panel III
NM: Nano meter
Rpm: Revolution per minute
SBP: Systolic blood pressure
SPSS: Statistical package for social sciences
T2DM: Type 2 Diabetic Mellitus
WC: Waist circumference
WHO: World Health Organization.
